# The frequency of candiduria in hospitalized patients with depressive syndrome

**DOI:** 10.12861/jrip.2014.27

**Published:** 2014-12-01

**Authors:** Ahmad Fakhri, Mojgan Navid, Zahra Seifi, Ali Zarei Mahmoudabadi

**Affiliations:** ^1^Department of Psychiatry, School of Medicine, Ahvaz Jundishapur University of Medical Sciences, Ahvaz, Iran; ^2^Department of Medical Mycology, School of Medicine, Ahvaz Jundishapur University of Medical Sciences, Ahvaz, Iran; ^3^Health Research Institute, Infectious and Tropical Diseases Research Centre, Ahvaz Jundishapur University of Medical Sciences, Ahvaz, Iran

**Keywords:** Candiduria, *Candida albicans*, Depressive syndrome

**Implication for health policy/practice/research/medical education:**

Our results shows that, the only long stay in hospital could not affect the prevalence candiduria in patients and other factors are need. In addition using anti depression drugs might be decrease the presence of *Candida* species in urinary tract.

Urinary tract infections (UTIs) are the most common infection among hospitalized patients and have been increased during the recent decades (1). Out of several microorganisms that are associated to UTIs including, yeasts are one of the most important organism that cause UTIs. The presence of *Candida* species in urine is considered as candiduria and it is varying from asymptomatic candiduria to clinical sepsis (2) and its incidence rate varies in different reports from different areas (2-5). Several predisposing factors are related to disease including; immunodeficiency, long term stay in hospital, hospitalized in intensive care unit (ICU) and neonatal intensive care unit (NICU), long term antibiotic therapy, elderly age, previous surgery, using indwelling catheters, urinary tract abnormalities, leukemia, bone marrow transplant and hemodialysis patients (4-9).

Depressive syndrome (DS) is a state of being depressed marked especially by sadness, inactivity, difficulty with thinking and concentration. In addition a significant increase or decrease in appetite and time spent sleeping, feelings of dejection, hopelessness, and sometimes suicidal thoughts or an attempt to commit suicide were seen. Although *Candida* species especially *Candida albicans* are as a part of human normal flora, candiduria is rarely presented in healthy individuals. Candiduria is most commonly caused by *C. albicans* followed by *C. glabrata* and *C. tropicalis*. However, non-*albicans* species; such as *C. tropicalis*, *C. glabrata*, *C. parapsilosis*, *C. lusitaniae*, *C. guilliermondii* and *C. krusei* was increased during last decades (3-5,10). The aim of present study was to identify and compare the presence of candiduria in patients with depressive syndrome with healthy individuals in two Salamat and Boustan hospitals affiliated to Ahvaz Jundishapur University of Medical Sciences (AJUMS).

In the present study all of the 131 (111 male and 20 female) patients with depressive syndrome hospitalized in mentioned hospitals were sampled. All patients were received several drugs including; antidepressant drugs (Amitriptylin), antipsychotic drugs (Haloperidol, Fentazin, Largactil), atypical antipsychotic (Risperidone, Olanzapine), central nervous system stimulants, monoamine oxidase inhibitors, selective serotonin reuptake inhibitors (Sertraline), anticholinergic (Artan, Ipridin) and mood stabilizer (Carbamazepine, lithium carbonate, sodium valproate). In addition 105 healthy individuals (79 male and 26 female) were also sampled as control. The age range of both patients and controls were 20-60 years.

Urine samples were collected in sterile urine bottles and transferred to medical mycology laboratory, department of medical mycology, AJUMS. 50μl of uncentrifuged urine samples was inoculated on CHROMagar Candida (CHROMagar Candida Co., France) and incubated at 37 °C for one week. All yielded yeasts were colony counted and identified based on morphology on CHROMagar Candida, production of germ tubes on fresh human serum, formation chlamydoconidia on cornmeal agar (Difco, USA) with 1% Tween 80 and ability to grow at 42-45 °C (11).

In the present study, 14 (13.3%) urine samples from control group were positive for *Candida* species, whereas only 3 (2.3%) of cases were positive. The colony counts in patients with DS were 20 CFU/ml of *C. glabrata* in a patient with 44 days, 60 CFU/ml of *C. albicans* in a patient with 60 days and 400 CFU/ml (40 *C. albicans* and 360 *C. krusei*) in another patient with 350 days stay in hospital. In control group the most common isolate was* C. glabrata* followed by *C. glabrata* (7, 50%), *C. albicans* (5, 35.7%) and *C. krusei* (1, 7.1%) and *Candida* species (1, 7.1%). The colony counts of yeasts in 4 control group was more than 20000 CFU/ml followed by in 6 control group 1500-8000 CFU/ml and 4 control group 150-660 CFU/ml. Totally the most common isolated yeasts from both groups were *C. glabrata* (44.5%) followed by *C. albicans* (38.9%), *C. krusei* (11.1%) and *Candida* species (5.5%).

In a study conducted by Zarei Mahmoudabadi *et al*. (4) out of 744 sample from hospitalized patients (different wards in Golestan hospital, Ahvaz) only 1.3% of positive candiduria was detected from psychiatry ward patients. Our results shows that only long stay in hospital could not affect on prevalence candiduria in patients and other factors are need. In addition using anti-depression drugs might be decrease the presence of *Candida* species in urinary tract.

## Authors’ contributions

AZM and AFdesigned and managed the research. MN collected urine samples, cultured and identified in laboratory. AZM analyzed data and ZS wrote draft manuscript. AZM and AF edited the final manuscript.

## Conflict of interests

The authors declare that there were no conflicts of interest throughout the study.

## Ethical considerations

Ethical issues (including plagiarism, data fabrication, double publication) have been completely observed by the authors.

## Funding/Support

The study was entirely carried out at the Department of Medical Mycology, Salamat and Boustan hospitals affiliated to Ahvaz Jundishapur University of Medical Sciences, and no funding was received.
